# Analysis of clinical efficacy of sacral magnetic stimulation for the treatment of detrusor underactivity

**DOI:** 10.3389/fneur.2025.1499310

**Published:** 2025-02-25

**Authors:** Huixian Pan, Chenhao Tang, Chen Song, Junhua Li

**Affiliations:** Department of Urology, Hangzhou Third People's Hospital, Hangzhou, China

**Keywords:** sacral magnetic stimulation, detrusor underactivity, urodynamic study, urination diaries, detrusor systolic pressure

## Abstract

**Objective:**

The objective of this study was to investigate the effectiveness and safety of sacral magnetic stimulation (SMS) in the management of detrusor underactivity (DU).

**Methods:**

We retrospectively analyzed 66 patients with detrusor underactivity treated at Hangzhou Third People’s Hospital from January 2020 to October 2024, divided into two groups (33 cases each). Both groups had confirmed detrusor underactivity via urodynamic studies. The control group received conventional treatment (medication, catheterization, bladder training), while the observation group received SMS therapy. Urination diaries, urodynamic parameters and self-rating anxiety scale (SAS) were collected before and after the 4-week treatment to evaluate SMS efficacy and safety.

**Results:**

All patients in the observation group completed the course of sacral magnetic stimulation without experiencing any serious complications. After treatment, the observation group showed a significant reduction in the number of daily urinations, nocturnal urinations, SAS score and residual urine volume (RUV) (*p* < 0.05) compared with the control group. There was no statistically significant difference in maximum cystometric capacity (MCC) (*p* > 0.05). However, improvements were observed in SAS score, Detrusor Pressure at Maximum Flow (Pdet), Bladder Contractility Index (BCI), Maximum urinary Flow Rate (Qmax) and Average Urinary Flow Rate (Qavg) (*p* < 0.05). The effective rate in the observation group was 78.78%, significantly higher than that in the control group (*p* < 0.05). Although there was a slight decrease in the effective rate during the 6-month follow-up, the difference was not statistically significant (*p* > 0.05).

**Conclusion:**

In conclusion, sacral magnetic stimulation therapy has demonstrated effectiveness in improving urinary function in patients with detrusor underactivity while maintaining a high level of safety.

## Introduction

Sacral Magnetic Stimulation (SMS) is an emerging non-invasive neuromodulation technique that operates on the principle of utilizing pulsed magnetic fields to modulate the activity of the sacral nerves. By inducing action potentials in the nerve fibers through the penetrative and adjustable properties of magnetic fields, SNMS can effectively regulate neural signaling pathways, thereby influencing the physiological functions of pelvic organs and lower limb control. Its working principle relies on the ability of magnetic fields to penetrate tissues and directly interact with neural structures, offering a non-invasive alternative to conventional electrical stimulation methods. The mechanism of action encompasses neural regulation, enhancement of pelvic floor muscle function, and pain relief through modulation of neural signal transmission (1–3).

Studies have demonstrated that SMS is more beneficial in enhancing both the maximum cystometric capacity and maximum urinary flow rate compared to nerve electrical stimulation (4). The sacral nerves, primarily originating from the sacral segments S2–S4, form a complex neural network that intricately controls bladder function through both parasympathetic and somatic pathways. The pelvic nerve, a key parasympathetic component, innervates the detrusor muscle to promote contraction and facilitate micturition, while the pudendal nerve, a somatic nerve, innervates the external urethral sphincter to enable voluntary control over urine release. SMS has the capacity to induce either excitation or inhibition of the detrusor, enhancing coordination between contraction and the sphincter muscle (5). Moreover, it plays a bidirectional regulatory role with varying parameter settings and initial bladder volumes, thus facilitating the restoration of normal urinary function (6). Currently, the majority of SMS studies have concentrated on the treatment of neurogenic bladder characterized by detrusor overactivity (DO). These studies have exhibited that SMS is capable of reducing intravesical pressure during the urinary storage phase and diminishing detrusor contraction following the urge to urinate (7, 8). Nonetheless, research regarding the application of SMS in the treatment of DU is scarce, and the results are inconsistent. DU is a prevalent urological condition, accounting for 11 to 40% of lower urinary tract symptoms and affecting both male and female individuals. It is characterized by a diminished contractility of the detrusor muscle, which results in prolonged bladder emptying or incomplete voiding within a normal time frame, as defined by the International Continence Society (ICS) (9). The management of DU continues to pose challenges, and SMS has introduced a novel approach and potential avenue for its treatment. This study aims to evaluate the efficacy of SMS in patients with DU and to explore its potential as a novel, safe, and effective clinical treatment strategy for this patient population.

## Materials and methods

### Clinical materials

A retrospective cohort study was conducted, enrolling a total of 66 patients with DU from January 2020 to October 2024 at the Department of Urology, Hangzhou Third People’s Hospital. Based on different treatment modalities, the patients were divided into a control group and an observation group. The enrollment criteria were as follows (10): (1) patients exhibiting clinical manifestations of dysuria, incomplete urination, or even urinary retention without surgical indications and with RUV greater than 220 mL, or patients with indwelling catheterization and bladder stoma, (2) Clinical presentation showed no urinary retention, but it was confirmed by urodynamic examination: male patients with BCI of less than 100 and a bladder outlet obstruction index (BOOI) of less than 20, and female patients with a maximum flow rate (Qmax) less than 15 mL/s and detrusor pressure at maximum flow (Pdet) less than 15 cm H_2_O (1 cm H_2_O = 98.0665 Pa), and (3) Patients voluntarily participated in and signed the informed consent form for this study. The exclusion criteria were as follows: (1) patients with severe heart, brain, lung, or other significant organ diseases, (2) patients with a history of severe kidney disease (e.g., hydronephrosis, renal calculus, nephritis), (3) patients with neurogenic bladder resulting from cauda equina or conus injury, (4) patients with mechanical urethral obstruction (e.g., prostatic hyperplasia, urethral stricture), (5) patients experiencing severe autonomic hyperreflexia during urination, (6) patients with sacral nerve electronic stimulator implantation, and (7) pregnant patients or those with severe urinary tract infections.

### Treatment

Before and after treatment, all patients underwent type B ultrasound to measure bladder RUV and urodynamic study (Laborie, Canada).

Patients in the control group received conservative medical therapy, which included pyridostigmine bromide (60 mg, three times daily) to enhance detrusor muscle contraction and tamsulosin (0.2 mg, once nightly) to reduce urethral resistance. Additionally, patients experiencing significant urination difficulty or having a high RUV underwent either indwelling or intermittent catheterization as needed.

In the observation group, patients received treatment with SMS (Magneuro100, Nanjing VISHEE Medical Technology Co., Ltd.) in addition to the treatment provided to the control group. The SMS procedure involved placing a figure-of-eight coil over the S3 nerve (midpoint between the sacrum and coccyx). Patients were seated and adjusted to ensure proper coil placement. Initial stimulation was delivered via a single pulse, and the S3 nerve response was assessed by observing toe flexion and anal contraction. Magnetic stimulation parameters were set as follows: intensity at 70–80% of the maximum, stimulation frequency at 10 Hz, intermittent stimulation with 20 s of continuous stimulation followed by a 2 s rest. Each treatment session was 10 min in duration and was administered twice daily, with an interval of more than 8 h between sessions, for 5 days per week over a total period of 4 weeks.

### Evaluation indexes

The urinary diary was kept for 3 consecutive days before and after treatment, including the number of urinations, the number of nocturnal urinations, and the maximum volume of urination. The mean value of each item in the 3-day urinary diary was used as the value of each index, respectively. Before and after treatment, the patients in both groups underwent a urodynamic study, which included the MCC, Pdet, BCI, Qmax, Qavg, and RUV. In addition, SAS was used to assess patient anxiety before and after treatment.

### Efficacy observation

The efficacy was assessed based on the changes in urodynamic study results and urination symptoms. The criteria for assessing efficacy were as follows: (1) Cured: significant improvement in symptoms of urination difficulty, RUV <20 mL, and, at the same time, for male patients, BCI > 100, or for female patients, Pdet >20 cm H_2_O and Qmax >15 mL/s; (2) Ineffective: no improvement in symptoms and no reduction in RUV, while for male patients, BCI < 100, and for female patients, Qmax <10 mL/s and Pdet <15 cm H2O; and (3) Effective: between cured and ineffective, with a reduction in RUV of 50% or more. The effective rate was defined as the percentage of patients who achieved either complete resolution (cured) or significant improvement (effective) of symptoms, calculated using the formula: (Number of cured cases + Number of effective cases)/Total number of cases×100%.

### Follow-up

The urinary diary and urodynamic indexes of the two groups were reassessed at 3 and 6 months after the completion of treatment to evaluate the effectiveness.

### Statistical analysis

The relevant data underwent statistical analysis utilizing SPSS 19.0 software. The normality of continuous variables was assessed using the Kolmogorov–Smirnov test. Normally distributed data were presented as mean ± standard deviation (Mean ± SD), while non-normally distributed data were presented as median and range Md (P1, P2). Group comparisons were carried out using the *t*-test, with significance set at *p* < 0.05.

## Results

The observation group included 33 patients (18 males, 15 females; mean age 38.6 ± 2.9 years; disease duration 15.3 ± 1.8 months). The control group had 33 patients (17 males, 16 females; mean age 38.9 ± 5.9 years; disease duration 14.9 ± 3.8 months). The general data of the two groups of patients were compared using statistical analysis, and the differences between the groups were not statistically significant (*p* > 0.05). This indicates that the groups are comparable, as demonstrated in Table 1.

**Table 1:** Basic condition of the two groups patients (mean ± SD).

	Observation group (*n* = 33)	Control group (*n* = 33)	*p* value
Gender (male/female)	18/15	17/16	0.859
Age (years old)	38.6 ± 2.9 (22–59)	38.9 ± 5.9 (27–63)	0.794
Course of disease (month)	15.3 ± 1.8 (1–19)	14.9 ± 3.8 (1–26)	0.586
Comorbid disease (*n*, %)			0.561
BPH (Benign Prostatic Hyperplasia)	7 (21.21%)	9 (27.27%)	
After BPH surgery	5 (15.15%)	7 (21.21%)	
Diabetic Cystopathy	10 (30.30%)	9 (27.27%)	
After the spinal cord operation	4 (12.12%)	5 (13.04%)	
After pelvic lymphadenectomy	4 (12.12%)	2 (6.06%)	
Idiopathic	5 (15.15%)	4 (12.12%)	

All patients completed the 4-week treatment. Efficacy results showed 11 cured, 15 effective, and 7 ineffective in observation group, the effective rate was 78.78%, significantly better than the control group (*p* < 0.05), as indicated in Table 2. Two patients experienced dizziness during magnetic stimulation, and three patients experienced waist pain after treatment, which all resolved spontaneously after rest. There were no complications such as infections, bleeding, or transient shock during the treatment.

**Table 2:** The curative effects of the two groups immediately after treatment and after 3 and 6 months (mean ± SD).

	Observation group (*n* = 33)			Control group (*n* = 33)		
	After treatment	3 months later	6 months later	After treatment	3 months later	6 months later
Cure (*n*)	11	10	8	4	2	1
Effective (*n*)	15	13	13	9	8	5
Noneffective (*n*)	7	10	12	20	23	27
Effective rate (%)	78.78%	69.70%	63.64%	39.39%	30.30%	18.18%
*p* value	*P*b < 0.001	*P*a:0.71	*P*a:0.174	*P*a < 0.001	*P*b: 0.438	*P*b: 0.057

*Pa: vs. observation group after treatment; Pb: vs. control group after treatment.*

Compared with the control group, the observation group exhibited significant reductions in the number of daily urinations, nocturnal urinations, SAS score, and RUV (all *p* < 0.05), as well as significant improvements in Pdet, BCI, Qmax, and Qavg (all *p* < 0.05) after 4 weeks of SMS treatment. However, there was no significant difference in MCC (*p* > 0.05) between the two groups. These results are summarized in Table 3.

**Table 3:** Comparison of urodynamic parameters and clinical data in the two groups before and after treatment (mean ± SD).

Item	Observation group (*n* = 33)		*p*	Control group (*n* = 33)		*p*
	Before treatment	After treatment		Before treatment	After treatment	
Daily urinations (times/ d)	24.19 ± 5.50	13.54 ± 4.62*	<0.001	24.67 ± 6.63^Δ^	22.8 ± 5.32	0.211
Nocturnal urinations (times/ d)	7.25 ± 1.73	2.93 ± 1.25*	<0.001	6.74 ± 2.35^Δ^	5.49 ± 3.36	0.085
BCI	81.64 ± 13.72	143.27 ± 22.35*	<0.001	82.32 ± 23.71^Δ^	92.41 ± 19.27	0.062
SAS score	65.82 ± 4.21	43.24 ± 8.72*	<0.001	68.47 ± 8.25^Δ^	64.29 ± 9.95	0.068
MCC (ml)	274.68 ± 95.36	289.45 ± 83.20**	0.505	285.35 ± 57.68^Δ^	290.55 ± 39.78	0.671
Pdet (cm H_2_O)	8.74 ± 3.21	19.15 ± 2.36*	<0.001	8.94 ± 2.89^Δ^	9.25 ± 1.75	0.600
Qmax (ml/s)	9.83 ± 1.23	13.58 ± 1.29*	<0.001	10.23 ± 0.72^Δ^	10.32 ± 1.42	0.746
Qavg (ml/s)	6.93 ± 1.37	10.67 ± 0.49*	<0.001	7.14 ± 1.03^Δ^	7.35 ± 0.75	0.347
RUV (ml)	235.49 ± 74.32	93.54 ± 21.29*	<0.001	242.45 ± 57.65^Δ^	235.17 ± 36.52	0.542

^Δ^: *p* > 0.05, vs. observation group before treatment; *: *p* < 0.05, vs. control group after treatment; **: *p* > 0.05, vs. control group after treatment.

After 6 months of follow-up, the therapeutic outcomes in the observation group were as follows: 8 patients were cured, 13 showed improvement, and 12 were ineffective. Although the effective rate declined compared with that at the end of the treatment, there was no statistically significant difference in efficacy between the end of treatment and the 6-month follow-up period (*p* > 0.05).

## Discussion

The etiology of DU can be classified into idiopathic, neurogenic, and myogenic. Since the detrusor contraction process relies on a complex neuromuscular pathway, any disruption along this pathway can impair detrusor contraction function. Previous studies have shown that contractility of the bladder detrusor muscle gradually decreases with age (11). Additionally, DU is frequently complicated by BOO, which often necessitates surgical interventions such as transurethral resection of the prostate (TURP) or transurethral resection of the bladder neck (TURB) (12). Prior studies have shown that while male patients with DU may achieve satisfactory short-term outcomes following TURP, the long-term results are often less than optimal (13). Many of these patients manage urinary problems by increasing abdominal pressure to promote urination, undergoing intermittent catheterization, or opting for cystostomy. However, these methods not only impact patients’ quality of life but also have a negative effect on their psychological well-being. In the treatment of DU, sacral nerve electrical stimulation is also utilized. However, the effectiveness of body surface electrical stimulation is limited and fails to achieve satisfactory curative results. Furthermore, internal sacral nerve electrical stimulation necessitates surgical implantation of electrodes, which can lead to complications such as infection, cerebrospinal fluid leakage, pain, and nerve root injury. Moreover, the treatment cost is expensive, thereby limiting its wide application in clinical practice to varying degrees (14).

SMS leverages Faraday’s law, where a changing magnetic field induces an electric field that generates a vortex current to stimulate nerves or muscles, aiding urination when targeting sacral nerves (15). SMS is effective for treating both DO and DU by modulating the activity of the sacral nerves. The key distinction in treating these conditions lies in the frequency of magnetic stimulation: for DO, lower frequencies (such as 10 Hz) are used to inhibit overactive detrusor contractions, while for DU, higher frequencies (such as 20 Hz) are applied to enhance detrusor contractility. This bidirectional regulatory capability allows SMS to address the distinct pathophysiological mechanisms underlying DO and DU, providing a versatile non-invasive treatment option (1, 8, 12). SMS alleviates DU through dual neuromodulatory mechanisms: (1) Pulsed electromagnetic fields penetrate pelvic tissues to induce eddy currents that selectively depolarize S2–S4 sacral nerve roots, enhancing parasympathetic efferent signaling and acetylcholine release at detrusor synapses (post-stimulation M3 receptor activity increased significantly) (16); (2) Repetitive stimulation upregulates brain-derived neurotrophic factor (BDNF) in Onuf’s nucleus, reversing axonal degeneration via TrkB-mediated survival pathways (17). Compared to conventional therapies, SMS demonstrates superior clinical utility: Unlike anticholinergics that suppress cholinergic transmission, SMS amplifies endogenous acetylcholine without systemic side effects (18); it has the same effect as electrical stimulation of the sacral nerve, producing a similar electric field but with less impedance and less attenuation of magnetic stimulation (19, 20); and it eliminates surgical risks associated with sacral neuromodulation [revision rate: 39% (21)]. Furthermore, SMS restores physiologic voiding, reducing catheter dependence compared to clean intermittent catheterization (CIC) (22), offering a non-invasive and disease-modifying alternative for DU management.

The study findings demonstrated a significant reduction (*p* < 0.05) in urination frequency, SAS score and RUV among patients with DU treated with SMS. Furthermore, compared to pre-treatment measures, there were improvements (*p* < 0.05) in Pdet, BCI, Qmax and Qavg. However, there was no significant change in MCC (*p* > 0.05). These results indicate that after undergoing a specific course of SMS treatment, neuromodulation induced by SMS can stimulate detrusor contraction, aiding in the recovery of urination function. This may be attributed to the coordinated functioning of the detrusor and sphincter, as well as the restoration of the micturition reflex pathway through SMS. Notably, the relative cystometric capacity does not increase. The reason for this may be that the main effect of SMS is to improve voiding function by enhancing the contractility and coordination of the detrusor muscle, rather than directly increasing the urine storage capacity of the bladder. Additionally, follow-up evaluations revealed that the response rate to SMS treatment gradually decreased over time, suggesting that repeated treatment sessions may be necessary to maintain therapeutic efficacy. Further investigation into the effects of repeated SMS treatments will be a focus of our subsequent studies.

## Conclusion

In summary, SMS demonstrated significant potential in improving urinary function in patients with DU, reducing urination frequency, nocturnal urination, and residual urine volume while enhancing detrusor contractility and urinary flow parameters. However, the study’s limitations should be noted. The small sample size restricted the statistical power and generalizability of the findings and did not allow for an effective assessment of the impact of different comorbidities on SMS treatment outcomes for DU. The short follow-up period limited the assessment of long-term efficacy and potential complications. Additionally, the retrospective design introduced selection and recall biases, as patients were not randomly assigned and data relied on existing records and patient recall. Finally, as a retrospective study, it was not possible to assess whether SMS could be used as an independent treatment for DU. Future research should address these limitations through larger, prospective, randomized controlled trials with extended follow-up periods to provide more robust evidence on the long-term efficacy and safety of SMS in treating DU.

## Data Availability

The raw data supporting the conclusions of this article will be made available by the authors, without undue reservation.
